# Characterization of mitochondrial dysfunction due to laser damage by 2-photon FLIM microscopy

**DOI:** 10.1038/s41598-022-15639-z

**Published:** 2022-07-13

**Authors:** Shagufta Rehman Alam, Horst Wallrabe, Kathryn G. Christopher, Karsten H. Siller, Ammasi Periasamy

**Affiliations:** 1 The W.M. Keck Center for Cellular Imaging, University of Virginia, Virginia, 22904 USA; 2 Departments of Biology and Biomedical Engineering, University of Virginia, Virginia, 22904 USA; 3 Advanced Research Computing Services, University of Virginia, Virginia, 22904 USA

**Keywords:** Optical techniques, Biological fluorescence

## Abstract

Mitochondria are the central organelles in cellular bio-energetics with key roles to play in energy metabolism and cell fate decisions. Fluorescence Lifetime Imaging microscopy (FLIM) is used to track metabolic changes by following the intrinsic co-enzymes NAD(P)H and FAD, present in metabolic pathways. FLIM records-lifetimes and the relative fractions of free (unbound) and bound states of NAD(P)H and FAD are achieved by multiphoton excitation of a pulsed femto-second infra-red laser. Optimization of multiphoton laser power levels is critical to achieve sufficient photon counts for correct lifetime fitting while avoiding phototoxic effects. We have characterized two photon (2p) laser induced changes at the intra-cellular level, specifically in the mitochondria, where damage was assessed at rising 2p laser average power excitation. Our results show that NAD(P)H-a2%—the lifetime-based enzyme bound fraction, an indicator of mitochondrial OXPHOS activity is increased by rising average power, while inducing changes in the mitochondria at higher power levels, quantified by different probes. Treatment response tracked by means of NAD(P)H-a2% can be confounded by laser-induced damage producing the same effect. Our study demonstrates that 2p-laser power optimization is critical by characterizing changes in the mitochondria at increasing laser average power.

## Introduction

Mitochondrion, the powerhouse of a cell is the central organelle in energy metabolism. It is the main driver for ATP generation via the Tricarboxylic Acid Cycle (TCA) and Oxidative Phosphorylation (OXPHOS), required by virtually all cellular and physiological processes. Mitochondria, while maintaining homeostasis in cellular bio-energetics, are also responsible for the initiation and execution of the programmed cell death or apoptosis via intrinsic pathways, hence play critical roles in cell-fate decisions. Defects in mitochondrial morphology, permeability, energy metabolism and oxidative stress, induce apoptosis by regulating the levels of Reactive Oxygen Species (ROS)^1^. Mitochondrial membrane potential (ΔΨm) *inter alia* also plays an important role in mitochondrial homeostasis by selectively eliminating dysfunctional mitochondria. Continuous changes in mitochondrial membrane potential result in the loss of cell viability^2^. Mitochondrial dysfunction and defective OXPHOS is increasingly being recognized in many disorders, particularly in cancer where the interplay between glycolysis and OXPHOS is altered^3,4^, facilitating growth, progression and metastases by modulating mitochondrial ROS and apoptosis^5–7^.

Fluorescence Lifetime Imaging Microscopy (FLIM) is a powerful non-invasive approach to investigate cellular metabolism in different pathophysiological states in real time, following the FLIM fingerprints of NAD(P)H (reduced nicotinamide adenine dinucleotide) and FAD (flavin adenine dinucleotide), the metabolic co-enzymes^8–11^, by infra-red multiphoton excitation. Both, NAD(P)H and FAD are endogenous, auto-fluorescent and are accepted biomarkers of metabolic states in different pathologies^12,13^. Only the reduced form of NAD(P)H and the oxidized form FAD are fluorescent. NAD(P)H signals originate from different cellular compartments, cytoplasm, mitochondria and nucleus and can be delineated based on their FLIM fingerprints at these subcellular locations^8^. NADH signals in the cell’s cytoplasm come from glycolysis and fermentation, in mitochondria from the pyruvate dehydrogenase complex, TCA and OXPHOS and from the nucleus by gene expression—the latter is not involved in cell’s metabolism^14,15^. FAD signals mainly come from the mitochondria^8,16^, as flavin mononucleotide (FMN) rests in complex I, while FAD is in complex II of the mitochondrial electron transport chain (ETC); both flavins belong to the approximately 10% covalently bound family of flavoproteins; many others form transient bonds catalyzing a variety of cellular reactions which are not involved in metabolism^17^.

The fluorescence lifetimes of NAD(P)H and FAD are sensitive to changes in their cellular micro-environment like pH, temperature, oxygen, viscosity, their conformational states, and proximity to quenchers^18^. These co-enzymes exist either in “free” or “enzyme-bound” states during cellular metabolic activity, and FLIM being a very sensitive tool, can discriminate between these states. Fitting of the fluorescence lifetime decays of NAD(P)H and FAD are typically based on a two-component exponential decay model^18^. The shorter (~ 0.4 ns) and longer (~ 2.4 ns) lifetimes of NAD(P)H represent the “free” and “enzyme-bound” components, respectively; the shorter (~ 0.12 ns) and longer (~ 3.38 ns) lifetimes of FAD represent the “enzyme-bound” and “free” components, respectively.

Mitochondrial oxidative phosphorylation (OXPHOS) activity consumes NADH (increased NADH-enzyme-bound fraction) and produces FAD (diminished FAD enzyme-bound fraction). Both the co-enzymes in their reduced (NAD(P)H and FADH2) and oxidized (NAD(P) + and FAD) forms participate in the cellular oxidation–reduction reactions critical for cell physiology^8,19^. Because of prominent changes of this fraction, NAD(P)H-a2% is a well-established metric to monitor OXPHOS activity by FLIM^8,9,19–23^.

Due to the observed lower intracellular levels/signals of FAD compared to NAD(P)H, it may be challenging to use FAD alone as a metabolic marker^16^. However, NAD(P)H due to its higher intracellular signal alone has been used in various studies to track metabolic activity^16,23,24^.

One-photon excitation of NAD(P)H can also be used at ~ 340 nm ultraviolet radiation^25–27^, which is potentially mutagenic and phototoxic to cells, therefore undesirable^25,28^—with multiphoton excitation at optimal power levels being a better alternative. In a seminal report, the damaging effects of pulsed NIR 730 nm excitation beam in comparison with the 1p-UVA absorption were investigated^25^. Increase in autofluorescence and its re-location to the nuclei were reported as indicators of cellular damage with 1p excitation. Similar changes were observed by 2p-excitation when either the number of scans or the average power levels were increased. Unlike our investigation, the cited study does not provide detailed experimental evidence for the increase in the autofluorescence or its nuclear re-location.

The mitochondria regulating cell-fate decisions has been the subject of study involving red and near-IR lasers in recent years^29–31^. In general, FLIM applications to investigate different disease models and treatment outcomes have grown over the years. It is therefore of vital importance to separate the potential laser-induced cell death from physiologically occurring changes. To answer this question, we have assessed the impact of irradiation by various laser average power intensities on mitochondria by FLIM.

Our FLIM methodology employs a 2-photon femto-second (Chameleon, Coherent) pulsed laser (680–1060 nm) at 5–7 mW power at the specimen plane^19,20,32^. The goal of this manuscript is to evaluate the damaging nature of increasing laser average power levels beyond the above optimized level on (1) mitochondrial metabolism and (2) to provide direct experimental evidence and mechanistic insight on phototoxicity induced changes particularly, in the mitochondria.

## Results

### Experimental approach

**Figure 1:**
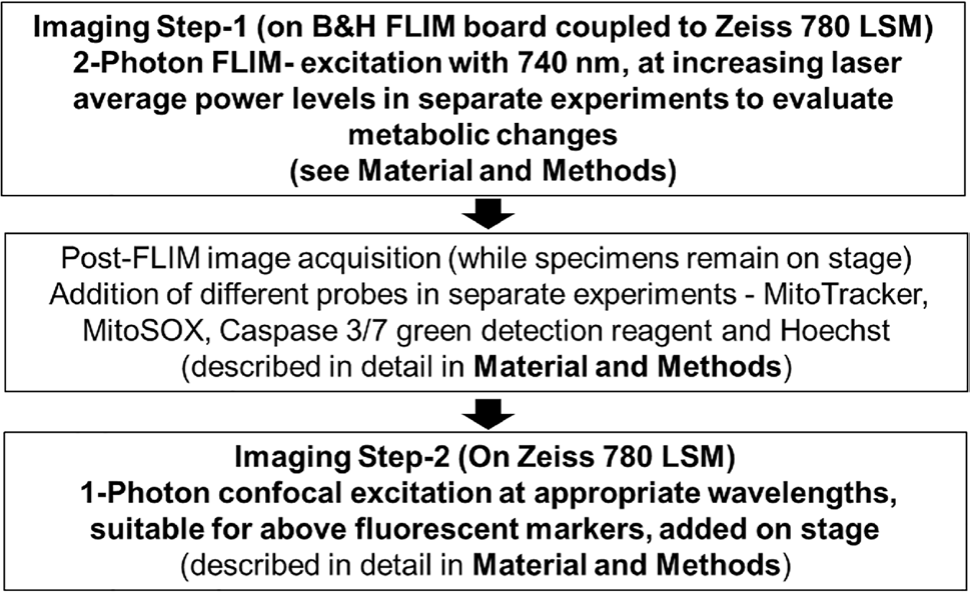
Flow Chart—2-step experimental strategy using a B&H FLIM board coupled to a Zeiss 780 LSM confocal, multiplexing multiphoton and confocal imaging. Following FLIM image acquisition (Step 1) of recorded X–Y–Z positions of different specimens with increasing laser average power in separate experiments, the specimens were labeled individually with different probes for mitochondrial viability and apoptosis and reimaged at identical X–Y–Z positions on the LSM Confocal side (Step 2) with probe specific 1-photon excitation under identical imaging settings to assess laser induced changes in mitochondria post FLIM.

The experimental strategy adopted to assess the effect of increasing laser average power output on mitochondria, is illustrated in Fig. 1. A two-step imaging sequence were applied, first 2-photon FLIM and then 1p confocal for all probes. Unlabeled cells were separately used for each of the listed 2-photon excitation average power (5.47 mW (2%), 12.88 mW (4%), 19.21 mW (6%) & 25.45 mW (8%)). Step-1: 2-photon FLIM was performed on 4 different FOVs of unlabeled cells with laser average power at 5.47 mW. The positions of the FOVs were recorded and saved. Step-2: After the desired incubation time on the stage, those saved FOVs irradiated by the 2p-pulsed laser were re-imaged on the confocal LSM side with probe specific 1p-excitation under identical imaging settings for the respective probes. These two steps were repeated for all the above listed 2p laser average power. On the LSM side a zoom factor 1.5 × was used versus a 2 × for FLIM in-order to assess the impact of laser irradiation just outside the perimeter of the FOV, previously irradiated by 2p pulsed laser. All experiments were repeated at least 2–3 times.

**Figure 2:**
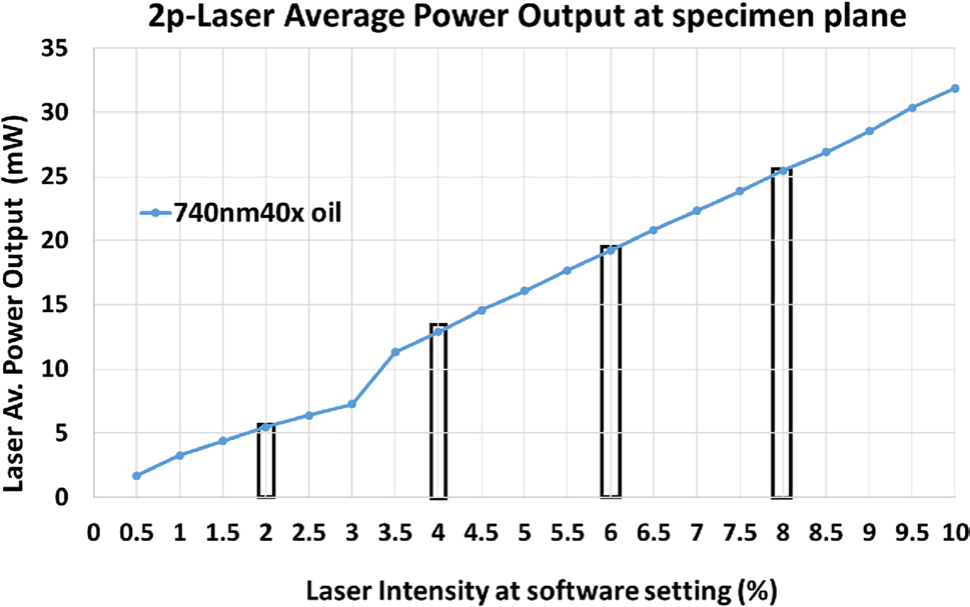
2p laser average power measured at the specimen plane. The average power of 2p laser at 740 nm was measured using a slide power meter (ThorLabs) with a 40 × oil, 1.3N.A lens. Laser average power at the specimen plane was correlated with the power setting of the software controller. The bars are the respective average power used in current investigation to assess 2p-laser induced mitochondrial damage (see the text for details).

Femto-second pulsed 2p lasers can elicit cytotoxic effects when imaging biological specimens^25^. It is therefore very important to measure the average power output of the femto-second pulsed 2p laser at the specimen plane. This was measured with a slide power meter for the 2p-740 nm (Fig. 2) laser on our Zeiss 780 confocal / multiphoton (MP) FLIM imaging system. Linear increase in average power output at the specimen plane was correlated with percentage (%) laser power level of the software controller (Fig. 2). 2p-laser induced transformations in the mitochondria were characterized with 740 nm excitation at the specimen plane for various average power levels as mentioned above.

### Transformations in mitochondrial morphology and increase in metabolic activity with increase in 2p-laser average power excitation in HeLa cells

**Figure 3:**
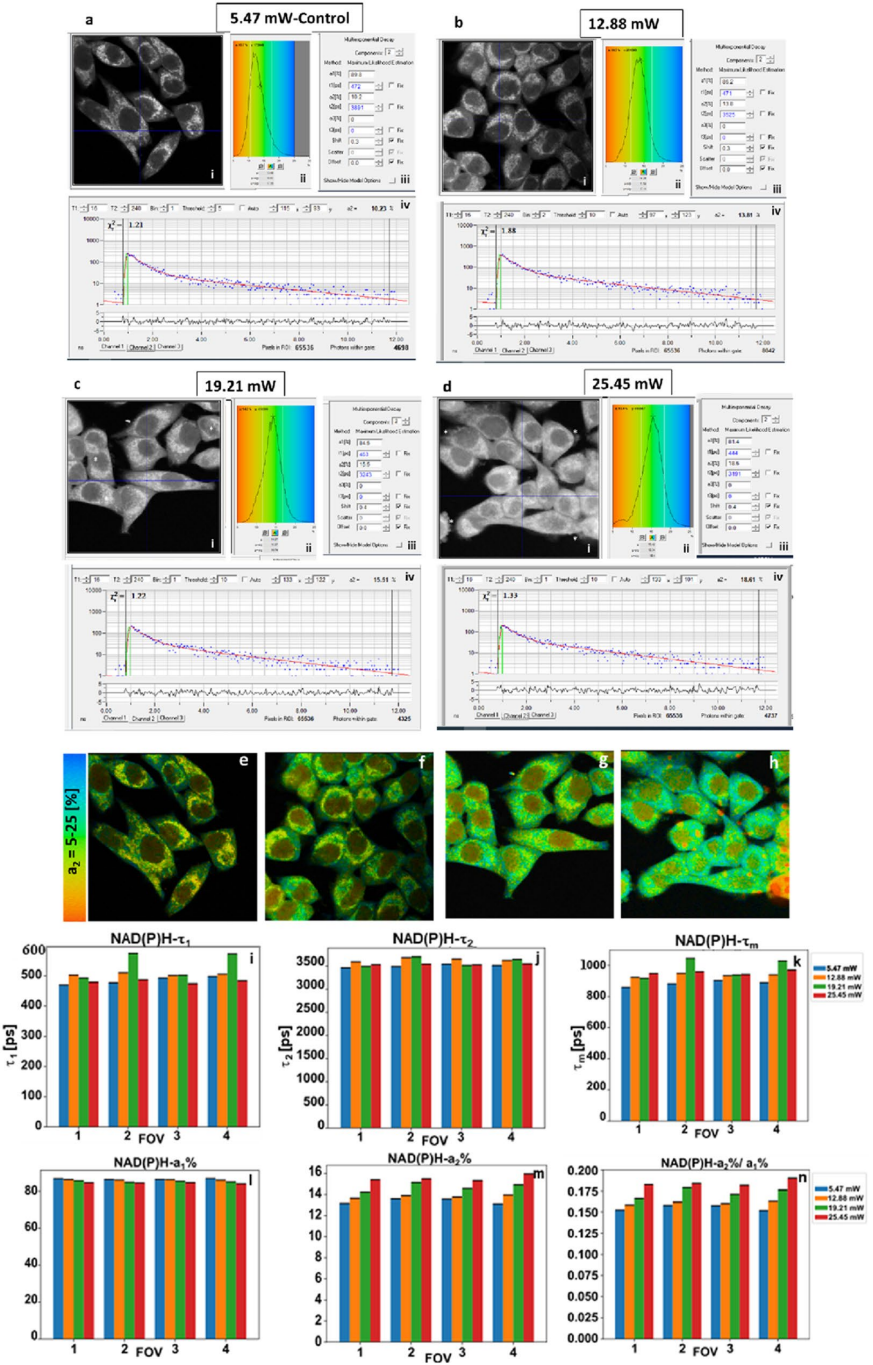
Mitochondrial OXPHOS metabolic marker NAD(P)H-a2% increases at rising 2p-740 nm laser average power in HeLa cells. Each panel (**a**–**d**) contains photon images (i) with corresponding NAD(P)H-a2% histogram (ii), lifetime parameters (iii) and the decay trace (iv). Panels (**e**–**h**) color-coded NAD(P)H-a2% images at different laser average power levels. Visually, mitochondrial morphology is clearly affected, confirmed later by mitochondrial viability markers (Figs. 4, 5 and 6). **(i**–**n)** Means (± SEM) of 4 merged FOVs (n = 70 cells) for several FLIM parameters. Except for natural cell variability, lifetimes τ_1_ (**i**) and τ_2_ (**j**) are unaffected by rising power, while NAD(P)H-a2%, the marker for OXPHOS metabolism increases (**m**), also affecting τ_m_, the amplitude-weighted-average lifetime (**k**). Rising NAD(P)-a2% also drives decreasing a1% (**l**) and increasing NAD(P)H-a2%/1% ratio (**n**).

Mitochondrial dysfunction and defective OXPHOS has been reported in cancer^5,33^. Correction of OXPHOS and induction of apoptosis via the mitochondrial pathway is a promising strategy in cancer treatment^5^. We have previously demonstrated by FLIM an increase in the NAD(P)H-a2%—the lifetime-based enzyme bound fraction and a widely used metric for mitochondrial OXPHOS activity^9,19,20,22,34^, to increase before the onset of doxorubicin induced apoptosis in prostate cancer cells and hence, established NAD(P)H-a2%-as an early predictor of treatment response^19^. Staurosporine-induced apoptosis and changes in NAD(P)H lifetimes with decrease in free-to-bound ratio by FLIM have also been previously observed^22^. Mitochondrial metabolism involves the pyruvate dehydrogenase complex (PDC), TCA and OXPHOS pathways. In the mitochondria, lifetime signals from the free form of NAD(P)H or its-a1% fraction come from the PDC and TCA whereas contributions of the enzyme-bound NAD(P)H-a2% originate from OXPHOS. Therefore, the ratios of enzyme-bound: free forms or NAD(P)H-a2%/a1% would indicate the overall status of the mitochondrial metabolic activity. Our current investigation shows transformations in mitochondrial morphology, and indicate an increase in the mitochondrial metabolic activity with excitation at increasing 2p-laser average power (Fig. 3) without any treatment or pharmacological interventions. Higher than optimal laser power can therefore be a confounding element and impossible to differentiate treatment from laser-induced changes. In Fig. 3, loss of discrete mitochondrial morphology can be seen in the representative photon images with higher average power of 19.21 mW (Fig. 3c) and 25.45 mW (Fig. 3d), in the form of cell shrinkage, rounding and some blebbing (denoted by* Fig. 3c(i),d(i)) as signs of morphological damage compared to 5.47 mW-Control (Fig. 3a(i))—our FLIM imaging standard. We have previously used NAD(P)H signals as a surrogate marker for mitochondrial morphology^19^. The representative color-coded images of NAD(P)H-a2% also showed loss of discrete mitochondrial morphology and rise in the NAD(P)H-a2%—enzyme bound fraction with rising laser average power (Fig. 3e–h). Since lifetime is independent of fluorophore concentrations, NAD(P)H τ_1_ and τ_2_ remained unaffected, except for some natural variability (Fig. 3i,j). τ_m,_ the amplitude weighted mean lifetime increased (Fig. 3k) driven mainly by NAD(P)H-a2% (Fig. 3m), mirrored by the decrease in the NAD(P)H-a1%, the free form (Fig. 3l). An increase in the NAD(P)H enzyme-bound to free ratio (Fig. 3n) also shows an overall increase in mitochondrial metabolic activity with the increase in the 2p-laser average power, confirming the confounding potential in treatment-based investigations measuring apoptosis.

### Loss of mitochondrial membrane potential (ΔΨm) with increase in 2p-laser average power excitation in HeLa cells

The experimental strategy used to assess the 2p-laser induced mitochondrial damage is illustrated in Fig. 1 and described in “Materials and methods”. Mitochondrial membrane potential (ΔΨm) is critical for mitochondrial homeostasis and a continuous rise or drop may result in the loss of cell viability^2^. To assess 2p-laser induced changes of mitochondrial ΔΨm we have used a MitoTracker CMXRos probe. Intensity changes of MitoTracker CMXRos are dependent on the physiological mitochondrial membrane potential (Fig. S1).

**Figure 4:**
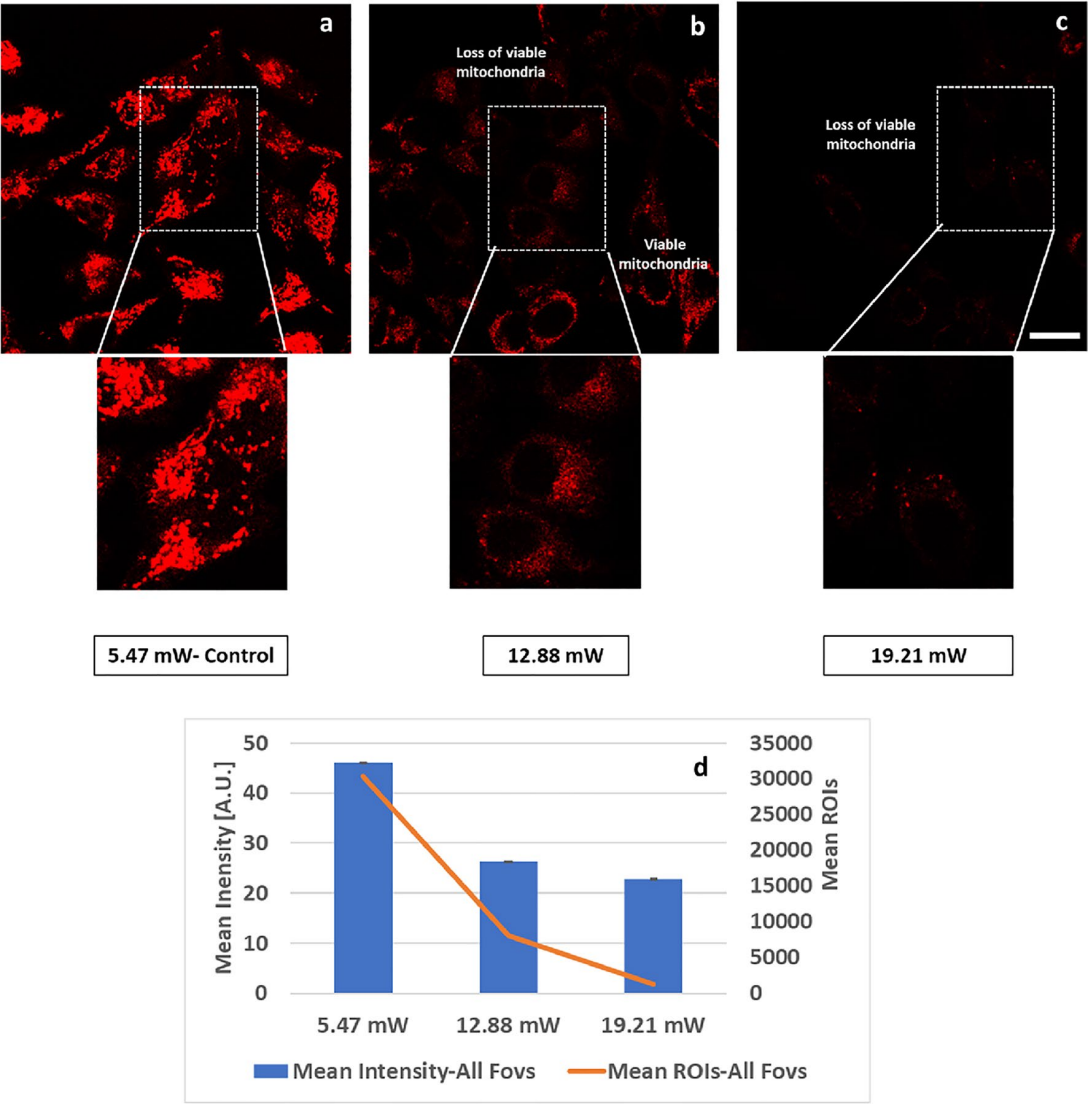
Loss of mitochondrial membrane potential (ΔΨm) at increasing 2p-laser power in HeLa Cells. (**a**) Representative confocal image of MitoTracker CMXRos signals (after FLIM acquisition at 5.47 mW, control laser power) showing viable mitochondria. (**b**) Loss of MitoTracker CMXRos confocal signal (after FLIM acquisition at 12.88 mW laser power) due to loss in mitochondrial ΔΨm. (**c**) After FLIM acquisition at 19.21 mW, MitoTracker CMXRos signals disappeared; it was pointless to track FOVs at 25.45 mW power. All confocal images were taken at identical conditions with 561 nm excitation at 0.3% power (Em 579–712 nm, PMT 630v). (**d**) Mean intensity (± SEM) bar plots and mean ROIs counts (2 × 2 pixel, orange line) from 4 FOVs show decrease in MitoTracker CMXRos mean intensity from 46 to 26.3 Gy-levels (8-bit image depth), a 42.84% decrease, and to 22.7—a 50.65% decrease. Mean ROI counts declined from 30,335 to 8,011.25 (73.63% decrease) and to 1,284.75 (95.77% decrease) after FLIM at 5.47 mW-Control, 12.88 mW and 19.21 mW respectively, demonstrating drop in signal with higher laser average power. Single factor Anova analysis was done for statistical significance between groups for mean intensity at alpha value of 0.05. The results were statistically significant with p value of < *0.0001*. Scale bar 20 μm.

Representative images and insets of MitoTracker CMXRos signals from viable mitochondria were seen with laser average power of 5.47 mW-Control (Fig. 4a), whereas loss of signals was observed after higher average power excitation at 12.88 mW (Fig. 4b) and 19.21 mW (Fig. 4c). Continuing to a higher power at 25.45 mW was pointless, considering the results at 19.21 mW. Some viable mitochondria outside of the FOV were still seen with 12.88 mW (Fig. 4b), which were completely lost with 19.21 mW irradiation (Fig. 4c). Our compiled results from all FOVs clearly demonstrate that excitation with higher average power resulted in the loss of physiological mitochondrial membrane potential leading to the loss of MitoTracker CMXRos labeling of the mitochondria (Fig. 4d). With respect to 5.47 mW-Control, a decrease of 42.84% with 12.88 mW and 50.65% with 19.21 mW average power in MitoTracker CMXRos mean intensity (Fig. 4d bar plot) was observed, validating that loss in mitochondrial membrane potential is critical in initiating mitochondrial metabolic response to higher laser average power induced damage (Fig. S2). Similarly, a decrease of 73.63% with 12.88 mW and 95.77% with 19.21 mW average power in MitoTracker CMXRos ROI counts (Fig. 4d orange line) with respect to 5.47 mW-Control was observed.

### Mitochondrial fragmentation with increase in 2p-laser average power excitation in HeLa cells

**Figure 5:**
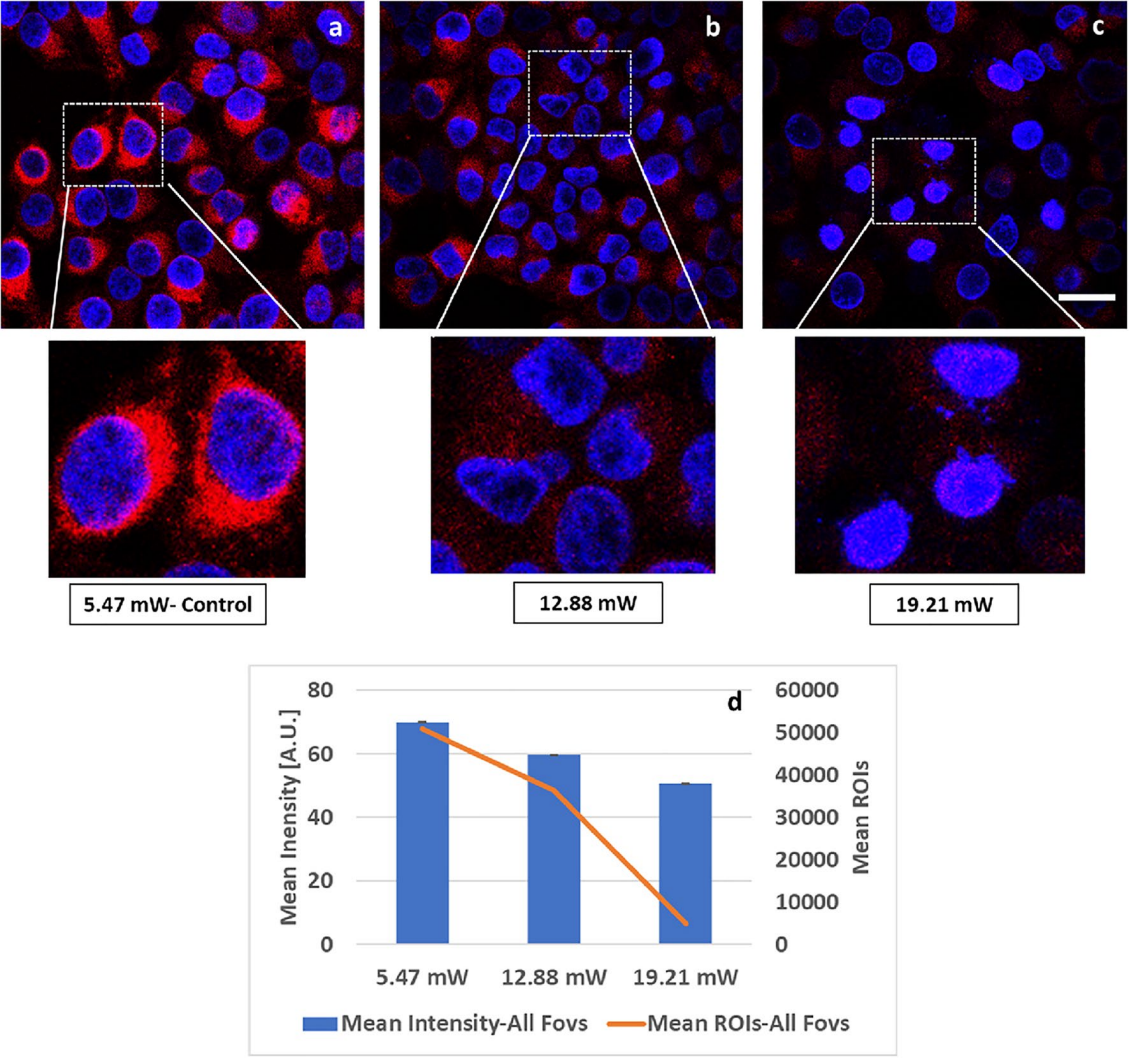
Mitochondrial fragmentation at increasing 2p-laser power in HeLa Cells. Representative confocal images of MitoTracker Red FM signals, imaged under identical conditions by confocal 594 nm excitation at 2.8% (Em-600–701 nm, PMT 850v) after FLIM acquisition at 740 nm. (**a**) Viable mitochondria after 5.47 mW–Control FLIM acquisition. (**b**,**c)** After higher FLIM laser power at 12.88 mW & 19.21 mW, increased mitochondrial fragmentation and loss in MitoTracker Red signal occurs. Hoechst nuclear stain. (**d**) Mean intensity (± SEM) bar plots and mean ROI counts (2 × 2 pixel, orange line) from 4 FOVs show a decrease in MitoTracker Red mean gray-level intensity (8-bit depth) from 69.86 to 59.71 (14.52% decrease) to 50.74 (27.36% decrease); and mean ROI counts (orange line) from 50,923.25 to 36,500 (28.32% decrease) to 4,971.66 (90.23% decrease) in ROI counts after FLIM with 5.47 mW-Control, 12.88 mW and 19.21 mW respectively, demonstrating drop in signal with higher laser average power. Single factor Anova analysis for mean intensity was done for statistical significance between groups at alpha value of 0.05. The results were statistically significant with p value of < *0.0001*. Scale bar 20 μm.

Mitochondrial fragmentation with 1 W femto-second pulsed laser has been reported^30^. To correlate our observations of morphological transformation seen in the mitochondria with the higher average power excitation (Fig. 3), we applied MitoTracker Red FM for visualization. Representative images and insets of MitoTracker Red signals from viable mitochondria were seen at 5.47 mW-Control (Fig. 5a). However, at higher average power of 12.88 mW (Fig. 5b) and 19.21 mW (Fig. 5c), we saw a loss of the MitoTracker Red signals. Our compiled results from all FOVs (Fig. 5d) demonstrate that excitation with higher 2p-laser average power resulted in the loss of discrete mitochondrial morphology. With respect to 5.47 mW—Control, a decrease in MitoTracker Red mean intensity of 14.52% and 27.36% (Fig. 5d bar plot); and a decrease in ROI counts of 28.32% and 90.23% (Fig. 5d orange line) was observed with 12.88 mW and 19.21 mW average power, respectively, corroborating our earlier observations in Fig. 3 of increased mitochondrial fragmentation, even without MitoTracker labeling. Since we have observed loss of mitochondrial membrane potential by the 2p-laser at higher power, it is quite possible that this has also contributed to the reduction in MitoTracker Red intensity. Nevertheless, both probes-MitoTracker Red and MitoTracker CMXRos show damage to the mitochondria after 2p-laser irradiation at higher power levels.

### Increase in mitochondrial oxidative stress with increase in 2p-laser average power excitation in HeLa cells

**Figure 6:**
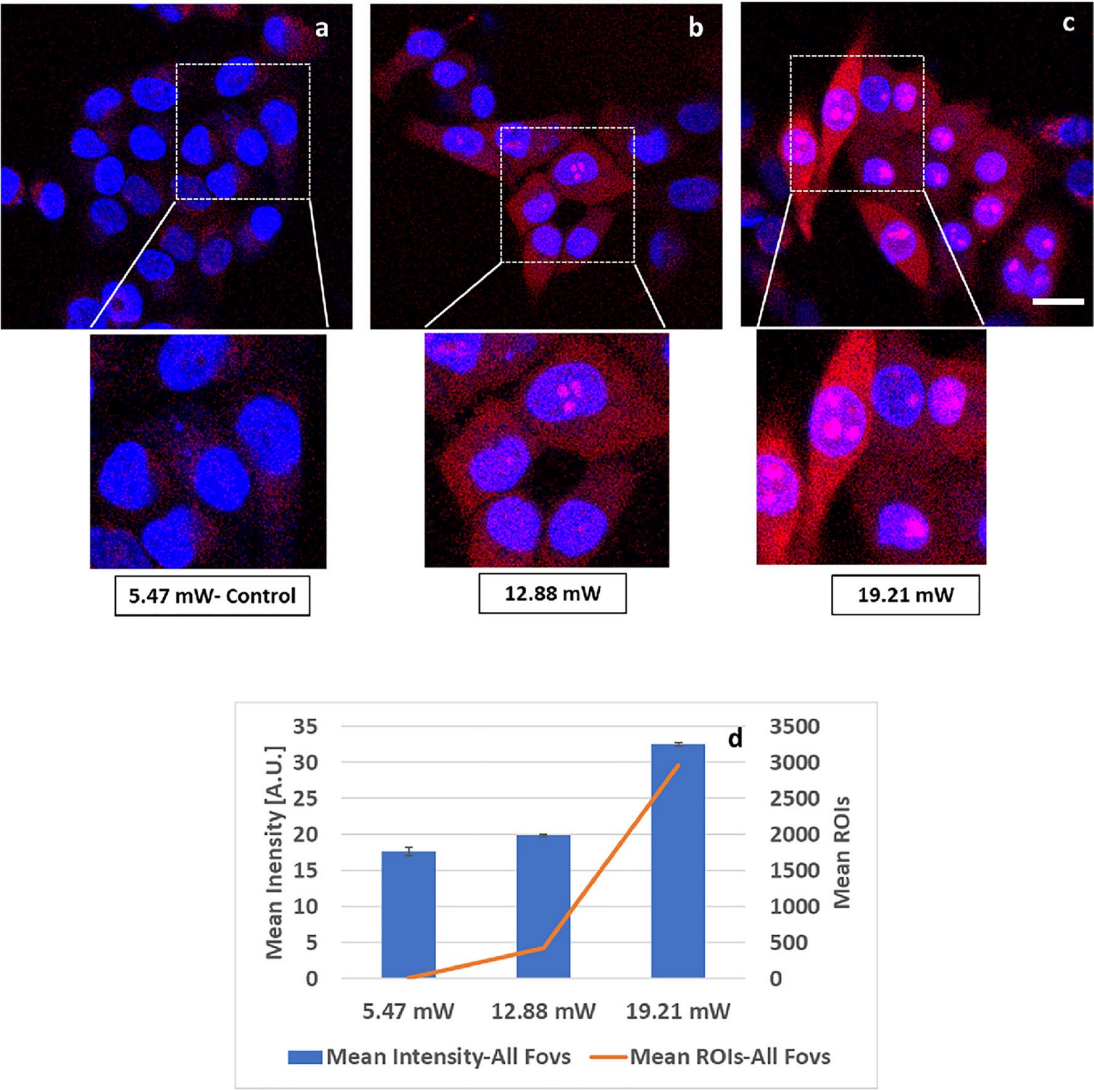
Increase in mitochondrial oxidative stress at increasing 2p-laser power excitation in HeLa cells. Representative confocal images of MitoSOX (mitochondrial ROS/superoxide) signal imaged under identical conditions with 514 nm at 10% (Em-551–683 nm, PMT 750v), after FLIM acquisition with 740 nm. All images were contrasted for brightness at the same settings to show the expected low-level ROS signal in cancer cells in the control image. Confocal MitoSOX signal after FLIM at laser average power of (**a**) 5.47 mW-Control and after increased FLIM laser power of (**b**) 12.88 mW and (**c**) 19.21 mW. The rising MitoSOX signal is indicative of oxidative stress not apparent in control (**a**). Hoechst nuclear stain was applied for nuclear localization. (**d**) Mean intensity (± SEM) bar plots and mean ROIs counts (2 × 2 pixel, orange line) from 4 FOVs show increase in MitoSOX Red mean gray-level intensity (8-bit depth) from 17.64 to 19.87 (12.6% increase) to 32.52 (84.3% increase); and mean ROI counts (orange line) from 10 to 427 (~ 40-fold increase) to 2964.75 (~ 300 fold increase) in ROI counts post FLIM with 5.47 mW-Control, 12.88 mW and 19.21 mW respectively-indicating increase in oxidative stress signal with higher laser average power. Single factor Anova analysis was done for mean intensity for statistical significance between groups at alpha value of 0.05. The results were statistically significant with p value of < *0.0001*. Scale bar 20 μm.

Femto-second near IR lasers can induce generation of highly reactive oxygen radicals or Reactive Oxygen Species (ROS) in biological specimens^29,30^. ROS induced oxidative stress-mediated cell death is a common mechanism seen in cells irradiated by near IR lasers^30^. MitoSOX Red is a selective live cell probe for detecting superoxide—the predominant ROS in the mitochondria. We have used MitoSOX Red to assess induction of oxidative stress with increase in average power excitation. Figure 6 shows representative images and insets of MitoSOX (mitochondrial ROS/superoxide) signals. Under identical imaging settings, a low-level signal from MitoSOX was detected at 5.47 mW-Control (Fig. 6a)—as expected in cancer cells; increased MitoSOX signals were observed at 12.88 mW (Fig. 6b) and 19.21  mW (Fig. 6c). Our data from all FOVs (Fig. 6d) show that with respect to 5.47 mW—Control, an increase in MitoSOX Red mean intensity (bar plot) of 12.61% and 84.30%; and tremendous increase in MitoSOX Red ROI counts (orange line) of ~ 40 and ~ 300 fold was observed with 12.88mW and 19.21mW average power, respectively, demonstrating that excitation with higher 2p-laser average power induces oxidative stress.

### Induction of apoptosis with higher 2p-laser average power excitation in HeLa cells

**Figure 7:**
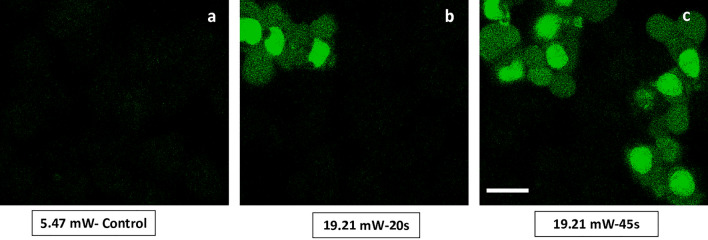
Induction of apoptosis with higher 2p-laser average power excitation in HeLa cells. Representative images show the signal of caspase 3/7 green detection reagent (CellEvents) imaged under identical conditions with confocal 488 nm at 2% (Em 493–579 nm, PMT 750v) after FLIM acquisition with 740 nm at (**a**) 5.47 mW–Control and after increased laser average power at (**b**) 12.88 mW and (**c**) 19.21 mW. No caspase 3/7 activation signal was detected after FLIM with (**a**) 5.47 mW–Control or with 12.88 mW (data not shown). However, after excitation with higher average power (**b**) 19.21 mW-20 s, caspase 3/7 activation signal and some apoptotic cells were observed which further increased with increase in the acquisition time (**c**) 19.21 mW-45 s. Scale bar 20 μm.

Oxidative stress-induced activation of apoptosis via intrinsic pathways, regulated by the mitochondria is well known^1^. Since we observed increase in MitoSOX signal with higher average power excitation, we further wanted to report on the induction of apoptosis in these irradiated cells. Caspase 3/7 green detection reagent (CellEvents) was used to assess induction of apoptosis with higher average power excitation. As a positive control, doxorubicin-treated HeLa cells were also imaged. (Fig. S3). No caspase 3/7 activation signal was detected with 5.47 mW-Control (Fig. 7a) or with 12.88 mW) (data not shown). Even though at 12.88 mW the apoptosis marker did not show changes, membrane potential changes, mitochondrial fragmentation and oxidative stress (Figs. 4, 5, 6) occurred, already indicating the deleterious effects of laser power at 12.88 mW. At higher average power levels of 19.21 mW some apoptotic cells were observed, which further increased in number with an increase in the photon acquisition time to 45 s vs 20 s (Fig. 7c), demonstrating the dosage and time-dependent nature of the pulsed femto-second laser in inducing oxidative damage and activation of apoptosis.

## Discussion

Infra-red femto-second pulsed lasers are widely used in fluorescence lifetime imaging of metabolic co-enzymes to understand cellular metabolism in normal and disease states. At higher power levels, the laser can induce phototoxic effects.

Mitochondria play key roles in bio-energetics and control cell-fate decisions. We investigated the very important question, whether the FLIM 2p-laser can damage mitochondria and if so, at what mW levels? Acquisition time of course is a companion variable and is usually the preferred way to avoid phototoxic cellular damage by increasing acquisition time and simultaneously reducing laser power levels. The challenge however remains to separate pharmacological interventions during imaging from laser induced and instrument related deleterious effects, as demonstrated in this manuscript.

In an earlier investigation, changes in the autofluorescence intensity, patterns and mean lifetime distribution of NAD(P)H coenzymes as a measure of 2p-NIR microbeam & 1p-UVA absorption induced photo-stress was probed^25^. Comparable to our results, the authors have shown that a similar threshold (2p-730 nm and 6 mW average power) with their imaging set up was safer for imaging NAD(P)H autofluorescence; and increase in autofluorescence and its re-location were reported as indicators of cellular damage with either repeating the number of scans or increasing the average power. Unlike the earlier study, this investigation provides direct experimental evidence of underlying mechanism of mitochondrial damage. As our research investigates mitochondrial dysfunction in diseases like cancer^4,19,20,32^ and Alzheimer’s by FLIM of NAD(P)H^24^, it is of utmost importance to determine that excitation with 2p-740 nm using our optimized standard conditions is safe and does not specifically damage mitochondria.

In the current study, we have investigated in a systematic manner the effect of 2p-740 nm laser induced transformations in the mitochondria by FLIM. As a second step, the extent of mitochondrial damage was investigated by 1-photon microscopy with several markers assessing mitochondrial viability (Figs. 4, 5, 6) and apoptosis (Fig. 7).

We have previously demonstrated that NAD(P)H-a2%-the lifetime-based enzyme bound fraction, an established and widely used metric^9,20,22,34^ for mitochondrial OXPHOS activity, to increase before the onset of apoptosis via the mitochondrial pathway^19^. Similarly, in the present investigation increase in the NAD(P)H-a2% indicating an increase in the mitochondrial OXPHOS activity, accompanied by the loss of discrete mitochondrial morphology was observed in response to excitation with higher laser average power in HeLa cells (Fig. 3). Morphological characteristics like cell shrinkage, rounding up and membrane blebs were also seen in some cells affected by the high average power (Fig. 3c(i),d(i),g,h), which was further confirmed by different mitochondrial probes to assess damage (Figs. 4, 5, 6).

Increase in the laser average power excitation from 5.47 mW (Control) to 12.88 mW and 19.21 mW resulted in the loss of mitochondrial membrane potential (Fig. 4) which affects cell viability. Not only that, it also resulted in mitochondrial fragmentation (Fig. 5) hence, affecting cellular metabolism. Phototoxicity generated by the increase in the laser average power was observed as oxidative stress (Fig. 6).

Many pro-death stimuli converge on the mitochondrial pathway leading to apoptosis^1,30,35,36^. Apoptosis via the mitochondrial intrinsic pathway is a long cascade with upstream and downstream events. Our results, indicate an increase in OXPHOS activity accompanied by the loss of discrete mitochondrial morphology, loss in mitochondrial membrane potential which eventually leads to changes in mitochondrial membrane permeability; mitochondrial fragmentation and increase in mitochondrial ROS-all provide mechanistic insights into the upstream events that result in the induction of apoptosis upon higher laser average power excitation. Activation of caspases is a downstream event in apoptosis. No caspase 3/7 activation signal was detected after excitation with 5.47 mW (Fig. 7) or with 12.88 mW in HeLa cells (data not shown). Apoptosis marked by the activation of caspase 3/7 was observed after excitation with the higher average power of 19.21 mW with 20 s photon acquisition time which increased further with increase to 45 s demonstrating the dosage and time-dependent nature of the pulsed 740 nm femto-second laser (Fig. 7).

Our results also provide evidence that our current FLIM set up using 740 nm-2% (software control) equivalent to 5.47mW average power at the specimen plane—used for several applications—is non-damaging to the mitochondria, used here as control to compare against excitation with higher laser average power to assess mitochondrial damage.

Different wavelengths in combination with different magnification objectives will produce different milli-watt levels at the specimen plane. To demonstrate this point, we have tested our instrumentation set-up with a combination of these 2 variables with a Thorlabs PM100D slide power meter (Fig. S4) and suggest that this is a required protocol for any FLIM imaging. Irrespective of the 2p-laser lines used for the excitation of NAD(P)H, our current contribution provides a guide on how to characterize and avoid 2p-laser induced mitochondrial damage. It specifically demonstrates how FLIM methodology, using femto-second pulsed 2p-laser with higher laser power at the specimen plane could damage mitochondria and trigger apoptosis.

No change in photon counts rates or photobleaching during FLIM image acquisition have been used to presume “no damage”. Also, DIC or brightfield images before and after the FLIM have been reported to assess phototoxicity^16^. This test alone may not be a true reflection of the earliest events of phototoxicity, as internal cues from within the cells are translated to external cues over time. The current investigation demonstrates that mitochondria, central to bio-energetics, are sensitive, labile organelles subject to photodamage. Our results provide evidence for earlier signs of mitochondrial photodamage, versus cell shrinkage, rounding and membrane blebbing which occur much later upon induction of apoptosis.

FLIM methodology is increasingly being used to understand cellular metabolism in different disease models, particularly in cancer, where cellular metabolism plays important roles in growth, progression, and metastases. FLIM methodologies are also being employed to characterize 2D vs 3D tumor models and assess treatment response^16,19,23,37^. The current investigation provides how optimization of the laser average power is critical for any FLIM based experimentation to separate treatment response from laser induced mitochondrial dysfunction.

Finally, to state the obvious, different instruments, different types of specimens and experimental objectives most certainly require different imaging settings. The main point and thrust of this paper therefore are, to amplify the need—not always followed in practice– firstly for optimization of all imaging conditions to avoid potential mitochondrial damage, which can be detected at earlier time points than apoptosis; secondly, to avoid potential ambiguities arising whether observed results are laser-based or treatment effects.

## Materials and methods

### Cell culture

Cervical cancer HeLa cells were grown in high glucose-Dulbecco’s Modified Eagle Medium (DMEM, Life Technologies) supplemented with 10% cosmic calf serum (Hyclone), 4 mM sodium pyruvate (Life Technologies) and 1% penicillin–streptomycin (Life Technologies). HeLa cells were maintained in cell culture incubator at 37 °C with 5% CO_2_. For all imaging experiments, cells were plated onto 25 mm round No.1.5 glass coverslips (Thermo Scientific), in their growth medium and grown to 70–80% confluence. All imaging experiments were done in DMEM Fluorobrite (Life Technologies) without serum.

### Laser average power output measurements

Power meter PM100D slide (ThorLabs) was used to measure the laser average power output at the specimen plane after 2-photon excitation with increasing laser intensities (%) in the software controller (Fig. 2). Laser average power output measured at the specimen plane with 40 × oil, 1.3 NA (same lens is used for imaging) for 740 nm at − 2% corresponded with 5.47 mW, 4% with 12.88 mW, 6% with 19.21 mW and 8% with 25.45 mW, used in this investigation.

### FLIM instrumentation, processing and analysis

Details on our FLIM instrumentation, processing and analysis is well described elsewhere^19,20,32^. In the present contribution, we have assessed the damaging effects on mitochondria with increasing laser average power output with 2-photon (2p) excitation by 740 nm ultrafast femto-second (150 fs) pulsed laser in HeLa cells. FLIM conditions were optimized, with varying acquisition times to achieve reasonable photon counts needed for reliable data fitting (data not shown). Table 1 summarizes the optimized FLIM imaging conditions used in this investigation. With higher laser average power output, photon acquisition time was reduced to sufficient photon count levels for reliable data fitting and to protect lifetime detectors from any damage.

**Table 1:** Conditions used for FLIM Imaging in this investigation.

740 nm laser average power output (mW) at the specimen plane with 40 × oil NA 1.3	740 nm laser intensity (%)—zen software controller (%)	Acquisition time (s) for FLIM	Pixel dwell time (μs)
5.47 mW (control)	2	45	1.58
12.88 mW	4	20	1.58
19.21 mW	6	20	1.58
25.45 mW	8	20	1.58

For image processing and analysis, the acquired FLIM images of NAD(P)H were fitted for 2-components with an incomplete-model using SPCImage software (v.8.3, Becker & Hickl). The offset and scattering were fixed to “0” and shift was optimized an χ^2^ close to 1. FLIM parameters were generated including photon images, τ_1_, τ_2_, τ_m,_ a_1_%, a_2_%, and χ^2^ for each pixel. As the dominant NAD(P)H signal corresponds to the mitochondrial morphology^19^, the mitochondrial Regions of Interest (ROI)s were thresholded by 1 × 1 pixels/ROI using the NAD(P)H photon image. The generated mitochondrial ROIs were then applied to the FLIM data to extract the data specific for these ROIs. The exported results from multiple FOVs were further analyzed as described elsewhere^20,32^ to produce, means and Bar charts with the standard error of the mean (± SEM).

### Reagents for live cell labeling of mitochondria

HeLa cells were labeled with different live cell probes for mitochondrial viability and apoptosis on stage maintained at 37 °C under humidified gas flow (5% CO_2_, 21% O_2_ and balanced with N_2_), in separate experiments. After the desired incubation time, those same FOVs irradiated by the 2p-pulsed laser with different average power levels were re-imaged on the confocal LSM side with probe specific 1p-excitation under identical imaging settings for the respective probes. On the LSM side a zoom factor 1.5 × was used versus a 2 × for FLIM in-order to also assess the impact of laser irradiation just outside the FOV directly irradiated by 2p pulsed laser. All live cell labeling and incubation were done in DMEM Fluorobrite without serum (Life Technologies). Mitochondrial staining with membrane potential dependent MitoTracker Red CMXRos at (Invitrogen) 50 nM for 20 min, MitoTracker Red FM (Invitrogen) 100 nM for 25 min, MitoSOX Red mitochondrial superoxide indicator (Invitrogen) 5 μM for 18 min; CellEvents caspase 3/7 green detection reagent (Invitrogen) 5 μM for 30 min and live nuclear stain Hoechst 33342 (Invitrogen) for 5–8 min incubation were used. Mitochondrial OXPHOS uncoupler CCCP (Sigma) at 50 μM for 20 min and anti-cancer drug doxorubicin at 1 μM and 5 μM, for overnight treatment were used in HeLa cells.

### CLSM

Identical imaging settings were used across different groups for Zeiss 780 confocal laser scanning microscopy (CLSM) of different probes to assess phototoxic effects of 2p-pulsed laser induced mitochondrial damage. For-MitoTracker Red CMXRos-Ex 561 nm at 0.3%, Em 579–712 nm, PMT 630v; MitoTracker Red FM-Ex-594 nm at 2.8%, Em-600–701 nm, PMT 850v; MitoSOX Red mitochondrial superoxide indicator-Ex-514 nm at 10%, Em-551–683 nm, PMT 750v; CellEvents caspase 3/7 green detection reagent-488 nm at 2%, Em 493–579 nm, PMT 750v and live nuclear stain Hoechst 33342 Ex—405 nm at 0.5–1%, Em—410–494 nm, PMT 700v with the same objective lens 40 × oil, 1.3NA.

### Statistical analysis

Results represented in Bar plots are expressed as mean ± SEM. Single factor Anova analysis at alpha value of 0.05 was used for statistical significance between groups showing changes in mean intensity for mitochondrial viability markers (Figs. 4, 5, 6).

## Supplementary Information

Supplementary Information.

## Data Availability

All data generated or analyzed in this study are included in this manuscript (and in the Supplementary Information).
