# Supplementary material for: Characterization of mitochondrial dysfunction due to laser damage by 2-photon FLIM microscopy
Source: Sci Rep. 2022 Jul 13;12:11938. doi: 10.1038/s41598-022-15639-z (PMC9279287; doi:10.1038/s41598-022-15639-z)
Supplement: Supplementary file 1 — Supplementary Information. [file 41598_2022_15639_MOESM1_ESM.pdf]

Supplementary Information

Title: Characterization of mitochondrial dysfunction due to laser damage by 2-photon FLIM microscopy

Shagufta R. Alam[1], Horst Wallrabe[1], Kathryn G. Christopher[1], Karsten H. Siller[3], Ammasi Periasamy[1,2]

[1]The W.M. Keck Center for Cellular Imaging, [2]Departments of Biology and Biomedical Engineering, [3]Advanced Research Computing Services, University of Virginia, Virginia, USA 22904

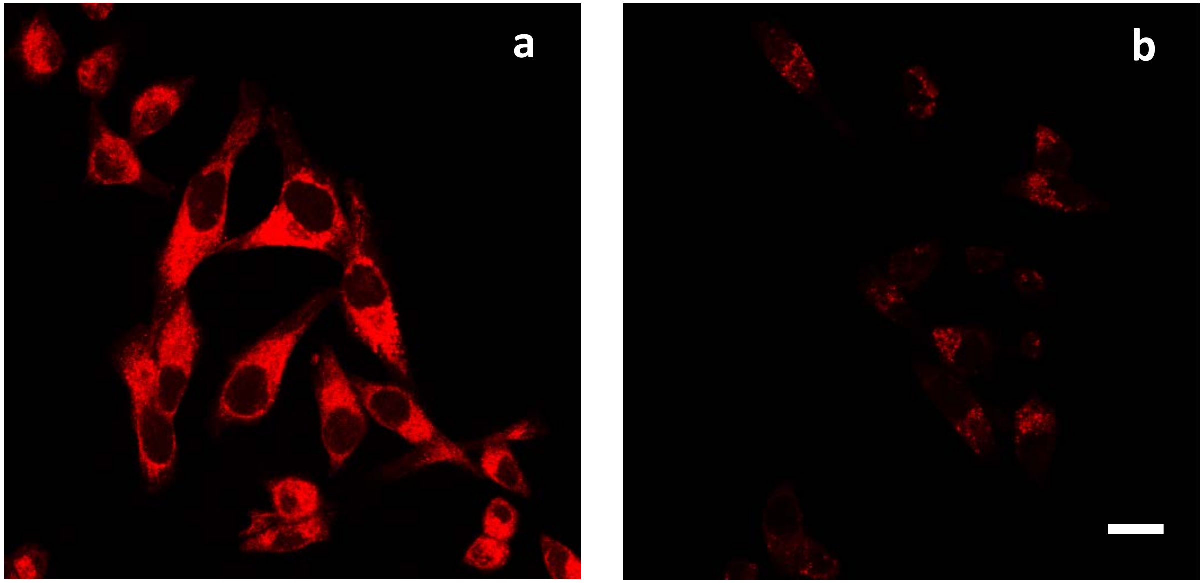

Figure S1. MitoTracker CMXRos labeling is specific and dependent on physiological mitochondrial membrane potential. To assess the specificity of the MitoTracker CMXRos based on the physiological mitochondrial membrane potential, HeLa cells were treated with mitochondrial OXPHOS uncoupler, Carbonyl Cyanide 3-Chlorophenylhydrazone or CCCP (Sigma) at 50 μM for 20 min. Control and CCCP treated HeLa cells were labeled with 100 nM of MitoTracker CMXRos for 30 min/37°C in Fluorobrite DMEM, fixed in 3.7% paraformaldehyde, mounted in Fluoromount G (Southern Biotech) and imaged on Zeiss 780 confocal/ multiphoton system. Identical imaging settings were used for control and CCCP treatment. Mitochondria labeled with MitoTracker CMXRos were observed in (a) control HeLa cells, whereas (b) the labeling was lost with the loss of mitochondrial membrane potential induced by the CCCP treatment, demonstrating that labeling of mitochondria by the MitoTracker CMXRos is dependent on the physiological mitochondrial membrane potential. Scale bar 20 μm.

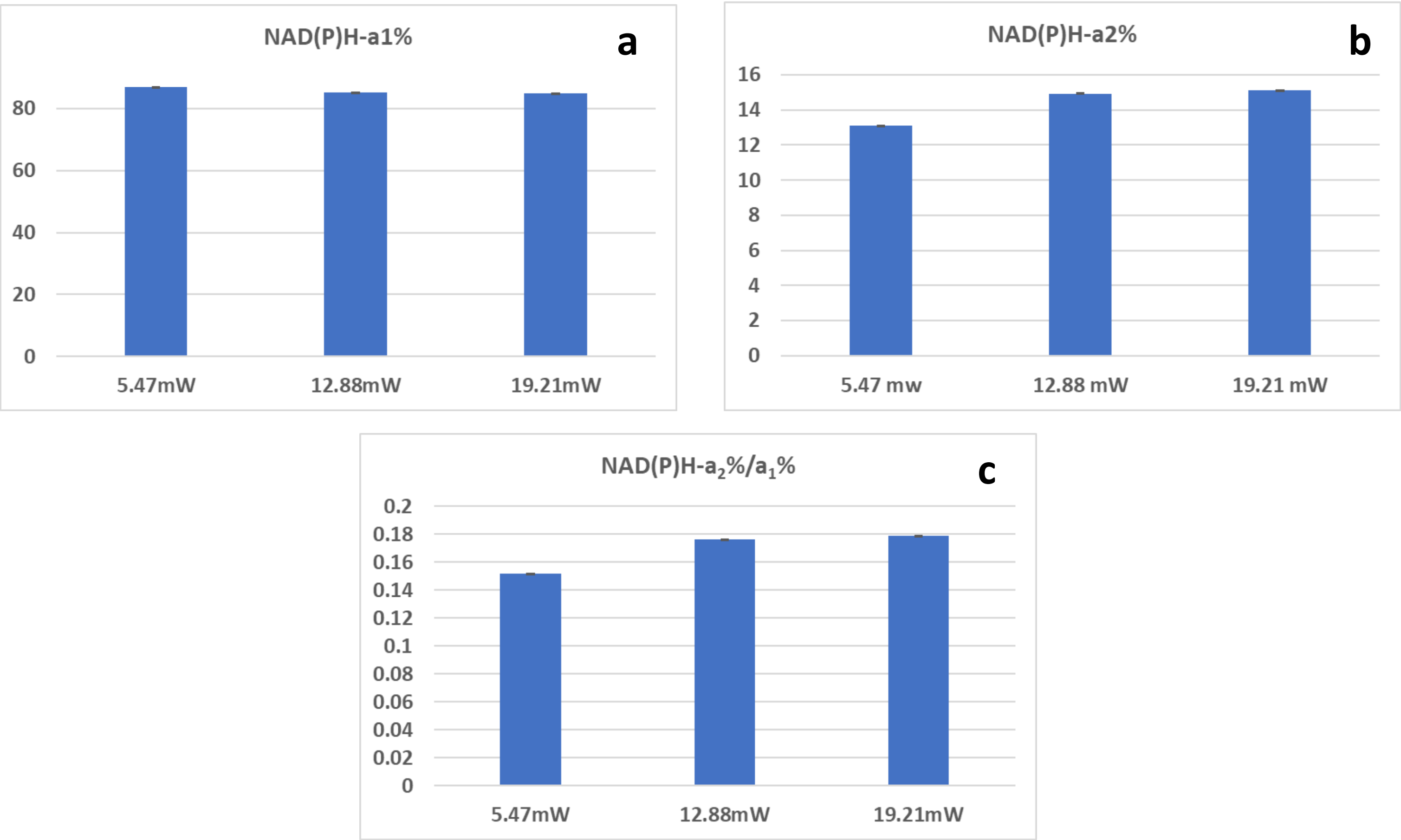

Figure S2. Bar graphs of Mean with S.E.M. corresponding to the FOVs (in Fig. 4) and representative to subsequent other FLIM experiments to probe mitochondrial damage- show combined results from 4 FOVs (n=~72cells) for NAD(P)H-a2%, the main marker of OXPHOS to be increasing from (b) 5.47 mW-Control to 12.88 mW and 19.21 mW. Increasing NAD(P)H-a2% is mirrored by decreasing NAD(P)H-a1% (a) and increasing NAD(P)H-a2%/NAD(P)-a1% ratio (c) as observed in Figure 2B(l-n). Single factor Anova analysis at alpha value of 0.05 was used for statistical significance between groups. The results were statistically significant with p value of 0.

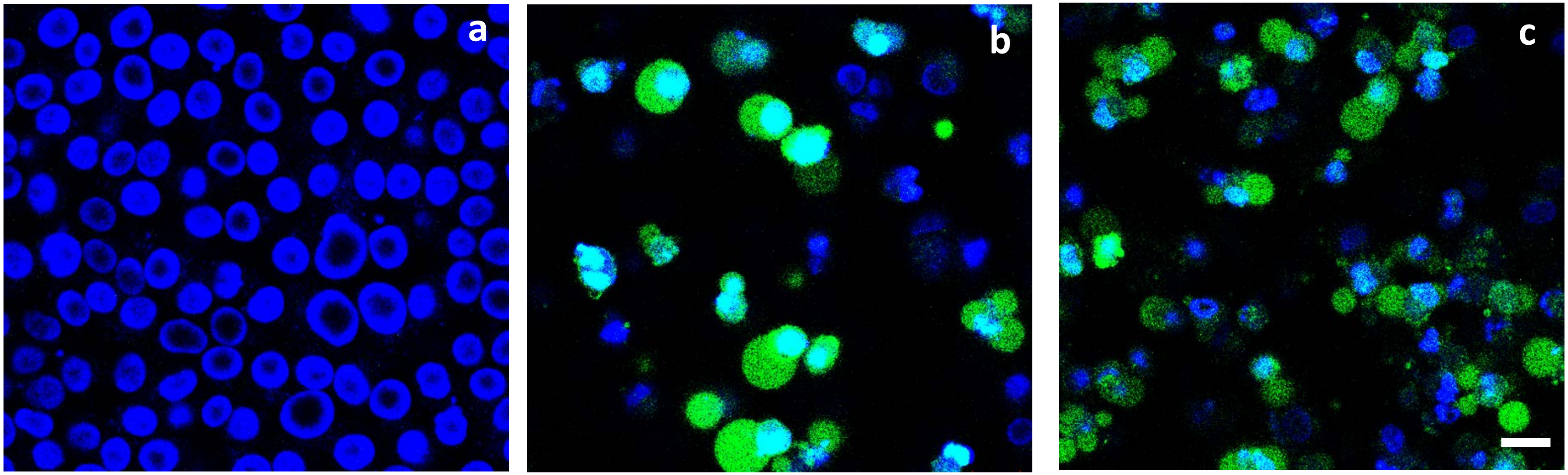

Figure S3. Caspase 3/7 green reagent confirms doxorubicin induced apoptosis in HeLa cells. To test the specificity of the caspase 3/7 green detection reagent (CellEvents) used in Fig 7, HeLa cells were treated with apoptogenic anti- cancer drug doxorubicin with (b) 1 μM and (c) 5 μM overnight. Control and doxorubicin treated cells were labeled with 5 μM caspase 3/7 green detection reagent (CellEvents) for 30 min/37°C and with Hoechst live nuclear stain. Imaging was done on Zeiss 780 confocal/ multiphoton system with identical imaging settings for control and treatment. Increase in signal from caspase 3/7 green detection reagent, marker for apoptosis was observed in doxorubicin treated cells whereas no signal was detected in the (a) control HeLa cells, demonstrating the specificity of caspase 3/7 green detection reagent in detecting caspase 3/7 activation and induction of apoptosis. Scale bar 20 μm.

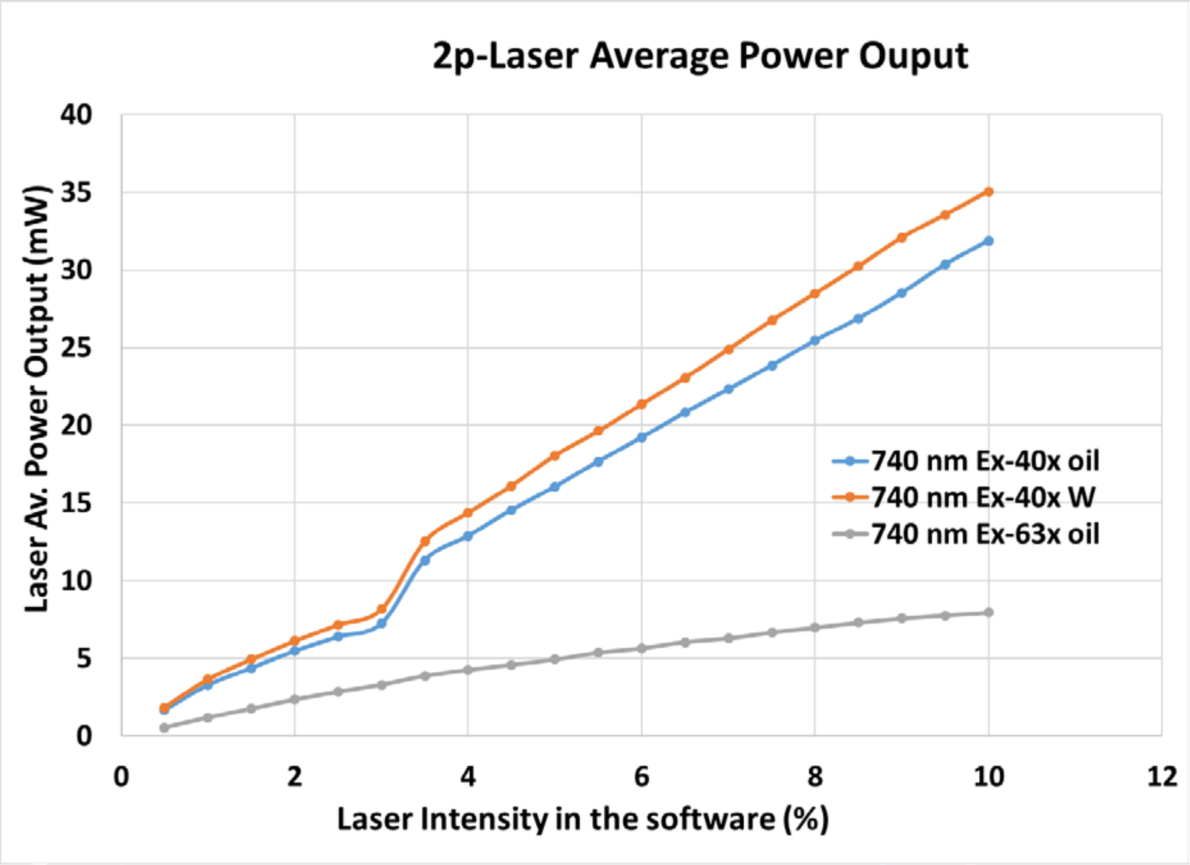

Figure S4. Comparison of the laser average power output at the specimen plane after excitation with the 2p 740 nm laser and different objective lenses. Thorlabs PM100D slide power meter was used for the measurement.
